# *SLCO1B3* promotes colorectal cancer tumorigenesis and metastasis through STAT3

**DOI:** 10.18632/aging.203502

**Published:** 2021-09-15

**Authors:** Lianghui Zhi, Lianmei Zhao, Xue Zhang, Wei Liu, Bo Gao, Feifei Wang, Xiaoran Wang, Guiying Wang

**Affiliations:** 1 Department of General Surgery, Hebei Medical University Third Affiliated Hospital, Shijiazhuang 050000, Hebei, China; 2 Scientific Research Center, Hebei Medical University Fourth Affiliated Hospital, Shijiazhuang 050000, Hebei, China; 3 The Second General Surgery, Hebei Medical University Fourth Affiliated Hospital, Shijiazhuang 050000, Hebei, China; 4 The First General Surgery, The 980 Hospital of PLA Joint Service Support Force, Shijiazhuang 050000, Hebei, China

**Keywords:** colorectal cancer, SLCO1B3, tumorigenesis, metastasis, STAT3

## Abstract

Solute carrier organic anion transporter family member 1B3 *(SLCO1B3)* is a gene that encodes an organic anion-transporting polypeptide (OATP) 1B3, a membrane-bound multi-specific transporter in hepatocytes. *SLCO1B3* was first reported in hepatocytes. Later, it was found that its expression is higher in colorectal cancer (CRC) than in the adjacent normal tissue. However, the role of *SLCO1B3* in CRC is not well elucidated. In this study, the correlation between *SLCO1B3* and the overall survival (OS) of CRC patients was evaluated using data from the GEO database. This study evaluated the relationship between *SLCO1B3* and the clinicopathological characteristics and prognosis of CRC patients. The effects of *SLCO1B3* knockdown, on human CRC cell proliferation, migration, and invasion *in vitro* and CRC tumorigenesis and metastasis *in vivo* were also examined. In addition, next-generation sequencing was used to identify *SLCO1B3* mediators. The results confirmed the association between *SLCO1B3* and poor OS of CRC patients, and *SLCO1B3* was identified as the top hub gene associated with the OS. The study showed that high *SLCO1B3* expression was associated with poor tumor differentiation, advanced disease stage, tumor invasion, lymph node metastasis, and poor OS. Next-generation sequencing revealed that *SLCO1B3* knockdown affected the expression of several genes involved in cancer invasion, metastasis, and DNA repair. Moreover, the western blot analysis showed that *SLCO1B3* knockdown downregulated p-STAT3, MMP-2, and MMP-9. In summary, we demonstrated that *SLCO1B3* acts as a novel carcinogen in the CRC that drives the CRC tumorigenesis and metastasis. *SLCO1B3* inhibitors, alone or in combination with current drugs, may have therapeutic benefits in CRC.

## INTRODUCTION

Colorectal cancer (CRC) is the fourth most commonly diagnosed cancer and the second leading cause of cancer-related mortality worldwide [1]. CRC is a highly aggressive malignancy with rapid progression. Its pathogenetic heterogeneity confers an escape mechanism to radiation, chemotherapy, and targeted therapies [2]. Despite recent advances in early diagnosis and treatment, the CRC prevalence and death rate in China have shown a significant upward trend over the past ten years [3], which sparked the need for effective management of CRC. Understanding the molecular mechanisms involved in CRC pathogenesis is key to discover new targets and therapeutic agents for CRC.

The *SLCO1B3* gene encodes an organic anion-transporting polypeptide (OATP) 1B3, a member of the liver-enriched OATP superfamily. The *OATP1B3* was first identified as a membrane-bound multi-specific transporter in hepatocytes responsible for the uptake of endogenous and xenobiotic substances [4, 5]. Later studies found that a variant of the liver-type *SLCO1B3* mRNA *(Lt-SLCO1B3)* is expressed in human cancer tissues and cell lines [6]. This cancer-type *SLCO1B3* mRNA *(Ct-SLCO1B3)* has a different transcription initiation site from the *Lt-SLCO1B3,* and its translated product *(Ct-OATP1B3)* mainly localized in the cytoplasm of cancer cells. Importantly, *Ct-SLCO1B3* was detected significantly higher than *Lt-SLCO1B3* in human colon cancer tissues [6]. However, the potential diagnostic and/or prognostic value and the functional role of *SLCO1B3* in human CRC remain unexplored.

This study evaluated the relationship between *SLCO1B3* and the clinicopathological characteristics and prognosis of CRC patients. We also investigated the function of *SLCO1B3* in human CRC cell proliferation, migration, and invasion *in vitro* and in CRC tumorigenesis and metastasis *in vivo*. The molecular mechanisms underlying the function of *SLCO1B3* in CRC were also explored.

## RESULTS

### Gene screening and the expression of SLCO1B3 in human CRC and its relationship with disease progression and survival

**Figure 1:**
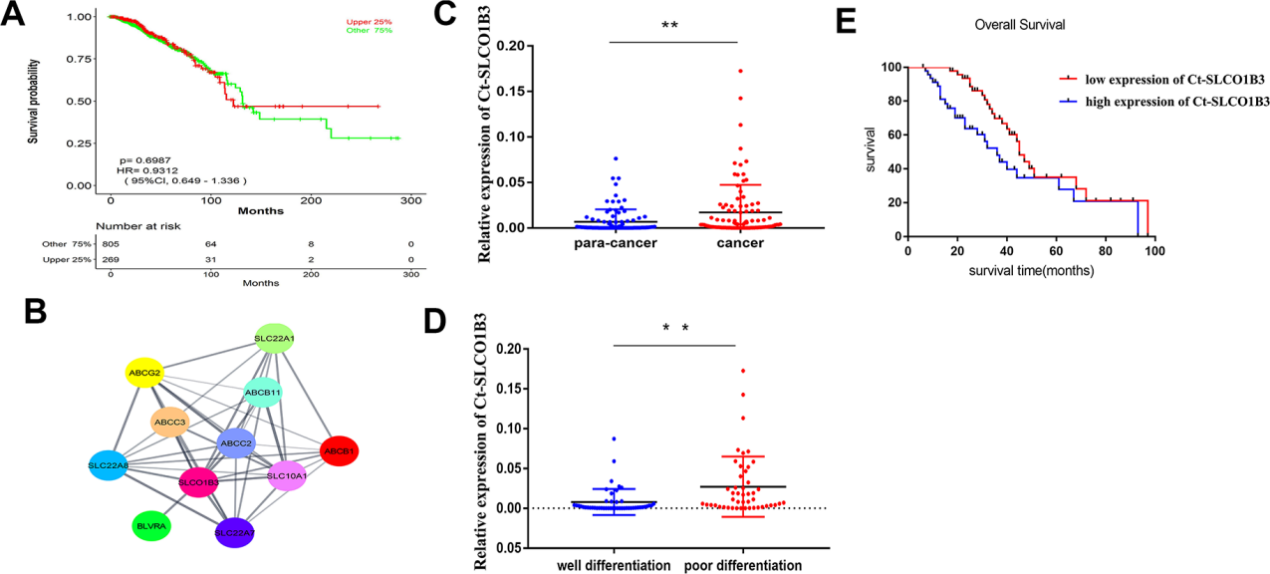
**The expression of *Ct-SLCO1B3* in human CRC and its correlation with tumor differentiation and survival.** (**A**) The relationship between OS and *Ct-SLCO1B3* expression in CRC patients was assessed by assessing the mechanism of *SLCO1B3* in colorectal cancer acceleration. GSE123734 dataset from the GEO database was downloaded that comprised of colorectal cancer samples. (**B**) Cytoscape and cytoHubba were used to catch the Hub genes based on GSE123734. As a result, *the SLCO1B3* gene with the highest score was considered a hub gene involved in the cell-matrix adhesion and MMPs pathway on GSE123734. (**C**) The *Ct-SLCO1B3* expression in cancer and adjacent normal tissues by qRT-PCR. n=96. **P < 0.01. (**D**) In poorly differentiated CRC, higher expression of *SLCO1B3* and in moderately well-differentiated CRC reduced expression of *SLCO1B3* has been noticed significantly. **P < 0.01. (**E**) Overall survival analysis of *SLCO1B3* expression in cancer tissues based one the collected clinical sample revealed that the high expression level of *SLCO1B3* was associated with a lower overall survival rate. The median *SLCO1B3* expression level was used as the cutoff for splitting high-expression and low-expression.

To further assess the mechanism of CRC acceleration, we downloaded the GSE123734 dataset from the GEO database, which comprised CRC samples. According to data in GSE123734, we found that a high expression level of *SLCO1B3* is associated with a lower overall survival rate (Figure 1A). Cytoscape and cytoHubba were used to catch the Hub genes. Consequently, the *SLCO1B3* gene with the highest score was considered a hub gene involved in the cell-matrix adhesion and MMPs pathway GSE123734 (Figure 1B). In the collected clinical samples compared with adjacent normal tissues, the human CCR tissues exhibited significantly higher *SLCO1B3* expression as revealed by qRT-PCR (P < 0.01, Figure 1C). *SLCO1B3* is highly expressed in poorly differentiated CRC, whereas reduced expression has been noticed in moderately well-differentiated CRC. The difference is statistically significant (P < 0.01, Figure 1D). Correlation analysis revealed that high tumorous *SLCO1B3* was associated with advanced disease stage, tumor invasion, lymph node metastasis, poor tumor differentiation, and low overall survival (Table 1 and Figure 1E). However, *SLCO1B3* expression was not associated with gender, age, tumor size, or tumor location in these patients (Table 1).

**Table 1:** The correlation between *Ct-SLCO1B3* expression and the clinicopathologic features of the 96 CRC patients.

**Clinicopathologic features**		**n**	***SLCO1B3***		**c^2^**	***P*-value**
			**Low**	**High**		
All		96	48	48		
Gender	Male	58	30	28	0.174	0.676
	Female	38	18	20		
Age	≥65	59	31	28	0.396	0.529
	<65	37	17	20		
Tumor size	≥6cm	41	21	20	0.043	0.873
	<6cm	55	27	28		
Tumor site	Left	59	31	28	0.396	0.529
	Right	37	17	20		
Tumor invasion	T1+T2	5	5	0	5.275	0.022
	T3+T4	91	43	48		
N stage	N0	45	33	12	5.880	0.015
	N1+2	51	15	36		
M stage	M0	89	47	42	4.909	0.027
	M1	7	1	6		
TNM stage	I+II	31	22	9	8.052	0.005
	III+IV	65	26	39		

Note: The median *Ct-SLCO1B3* expression level was used as the cutoff for splitting high-expression and low-expression tumors. The Chi-square test was used to analyze the correlation between the clinicopathologic features and *Ct-SLCO1B3* expression level. *P* < 0.05 indicates statistical significance.

### SLCO1B3 expression in CRC cell lines and select effective interfere sequence

**Figure 2:**
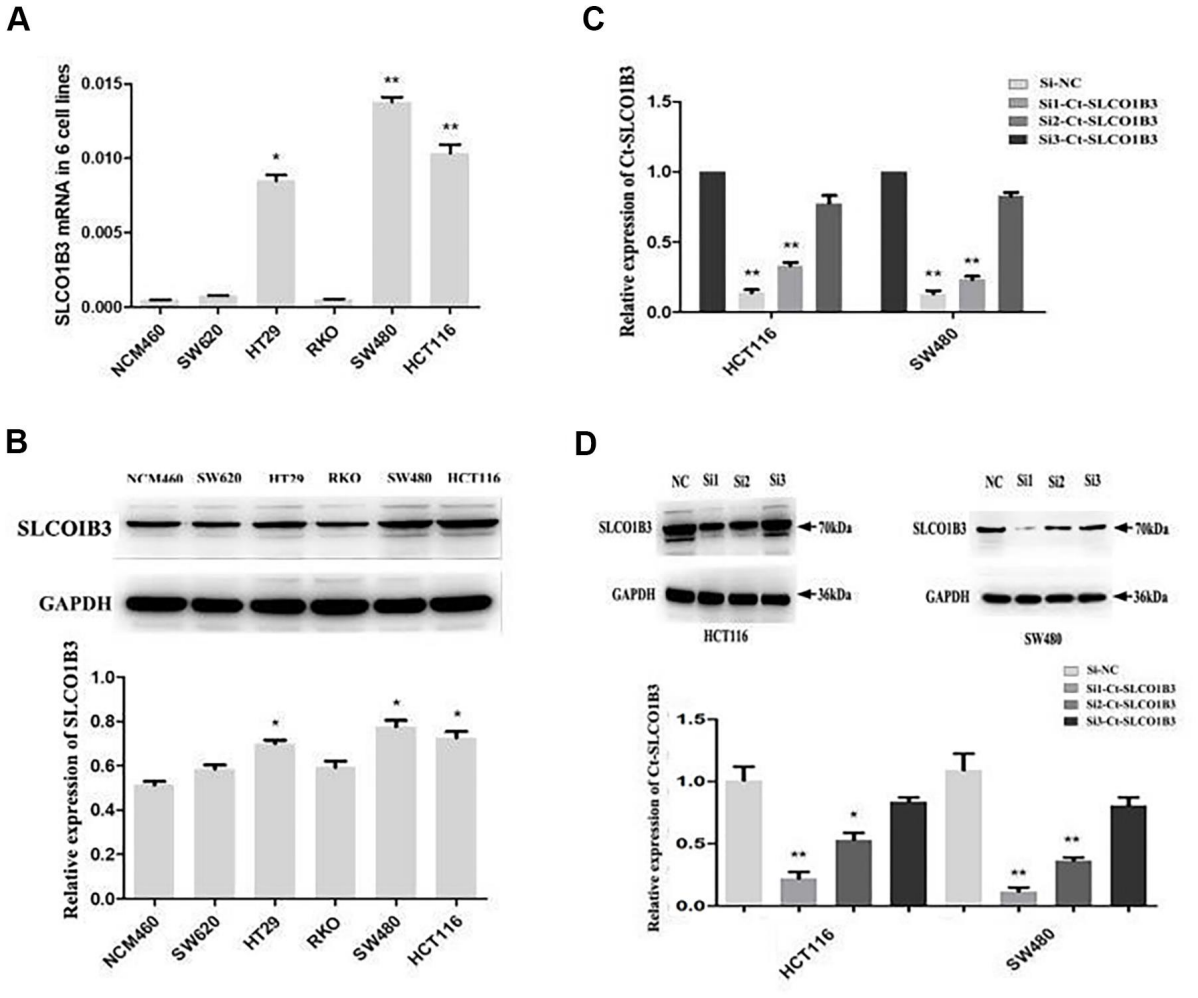
***Ct-SLCO1B3* expression in CRC cell lines and select effective interfere sequence.** (**A**, **B**) The mRNA (**A**) and protein (**B**) levels of *Ct-SLCO1B3* in the NCM460 normal human colon epithelial cell line and the SW480, SW620, HT29, HCT116, and RKO human CRC cell lines by qRT-PCR and western blot analysis, respectively. n=3; *P < 0.05, **P < 0.01 vs. NCM460. (**C**, **D**) HCT116 and SW480 cells were transiently transfected with *si1-Ct-SLCO1B3*, *si2-Ct-SLCO1B3*, *si3-Ct-SLCO1B3*, or si-NC. (**C**) The *Ct-SLCO1B3* mRNA levels by qRT-PCR. (**D**) The *Ct-OATP1B3* protein levels by western blot analysis. n=3; *P < 0.05, **P < 0.01 vs. si-NC. NC=si-NC, Si1=s*i1-Ct-SLCO1B3*, Si2=*si2-Ct-SLCO1B3* and Si3=*si3-Ct-SLCO1B3*.

We found that high *SLCO1B3* expression in human CRC tissues was associated with advanced disease, tumor invasion, lymph node metastasis, and poor patient survival. We investigated the role of this gene in CRC tumorigenesis *in vitro* and *in vivo*. We examined *SLCO1B3* expression in the NCM460 normal human colon epithelial cell line and the SW480, SW620, HT29, HCT116, and RKO human CRC cell lines. The HCT116, HT29, and SW480 CRC cell lines showed significantly higher *SLCO1B3* expression than the NCM460 cell line (Figure 2A, 2B). To evaluate the function of *SLCO1B3* in human CRC cell proliferation, migration, and invasion *in vitro*, we generated HCT116 and SW480 cells transiently transfected with *si1-SLCO1B3*, *si2-SLCO1B3*, *si3-SLCO1B3*, or si-NC. The *SLCO1B3* expression was effectively reduced with *si1-SLCO1B3* or *si2-SLCO1B3*, but not with si3-*SLCO1B3* transfection (Figure 2C, 2D). Subsequently, the HCT116 and SW480 cell lines were used to perform the followed research, transfected by *si1-SLCO1B3* and *si2-SLCO1B3*.

### SLCO1B3 knockdown inhibits human CRC cell proliferation, migration, and invasion *in vitro*

**Figure 3:**
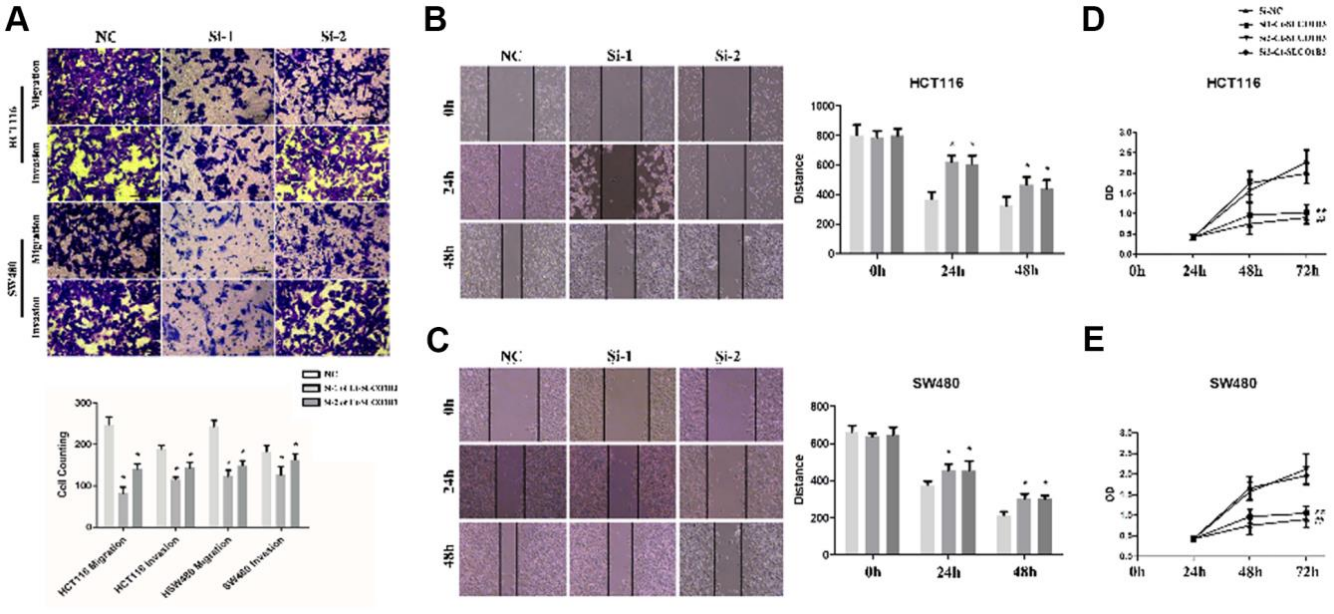
***Ct-SLCO1B3* knockdown inhibits human CRC cell proliferation, migration, and invasion *in vitro*.** (**A**) Cell migration and invasion abilities evaluated by the Transwell assays. The cells that had migrated to the lower surface of the membrane were counted after 48-hour incubation. n=3; *P < 0.05, **P < 0.01 vs. NC. (**B**, **C**) The scratch-wound healing assay evaluated cell migration ability. The width of the wound bed (distance) was measured at 0, 24, and 48 hours after the creation of the scratch wound. (**D**, **E**) Cell proliferation evaluated by the MTS assay. n=3; *P < 0.05, **P < 0.01, ##P < 0.01 vs. si-NC or NC. NC=si-NC.

Compared with the corresponding control cells, *SLCO1B3*-silenced HCT116 and SW480 cells exhibited significantly reduced migration and invasion abilities as indicated by the Transwell and wound healing assays (P < 0.05; Figure 3A–3C). In addition, the MTS assay revealed decreased proliferation of the *SLCO1B3*-silenced cells than their corresponding control (P < 0.05, Figure 3D, 3E).

### The effects of SLCO1B3 knockdown on CRC tumorigenesis *in vivo*

**Figure 4:**
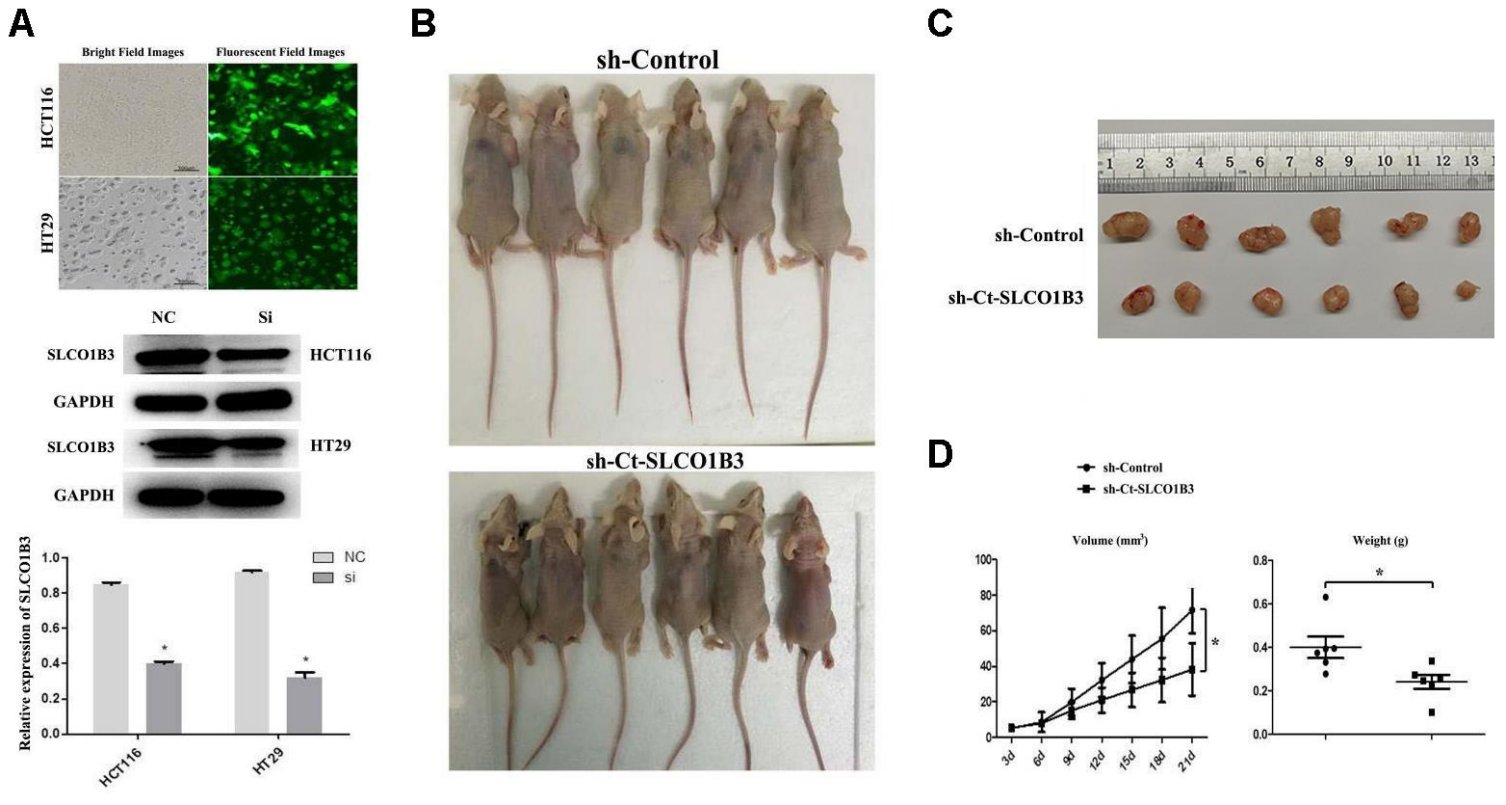
**The effects of *Ct-SLCO1B3* knockdown on CRC tumorigenesis *in vivo*.** (**A**) Characterization of the HCT116 and HT29 cells stably transfected with a lentiviral vector carrying *sh-Ct-SLCO1B3* or sh-Control. Bright and fluorescent field cell images (upper panel) and western blot analysis results on *Ct-OATP1B3* protein expression (middle and lower panels) are shown. n=3, *P < 0.05 vs. sh-Control. (**B**) Photos of mice bearing subcutaneous tumors. (**C**) Photos of tumors harvested from the mice. (**D**) The change in tumor volume with time and the weight of the harvested tumors. *P < 0.05.

We investigated the role of *SLCO1B3* in CRC tumorigenesis in a mouse xenograft model and subsequently generated HCT116 and HT29 cells. They were then stably transfected with a lentiviral vector carrying *sh-SLCO1B3* or sh-Control. *SLCO1B3* knockdown was confirmed with western blot analysis (Figure 4A). Female BALB/c nude mice received HCT116 cells with stable *SLCO1B3* knockdown *(sh-SLCO1B3)* or an equal number of negative controls HCT116 cells (sh-Control) by subcutaneous injection into the front flank (n=6 per group). The mice were euthanized 21 days after the inoculation. We found that xenograft tumors derived from *SLCO1B3*-silenced HCT116 cells grew significantly slower than those derived from control cells (P < 0.05). This resulted in smaller and lighter tumors after three weeks of growth (Figure 4B–4D).

### SLCO1B3 activates STAT3 in human CRC cells

**Figure 5:**
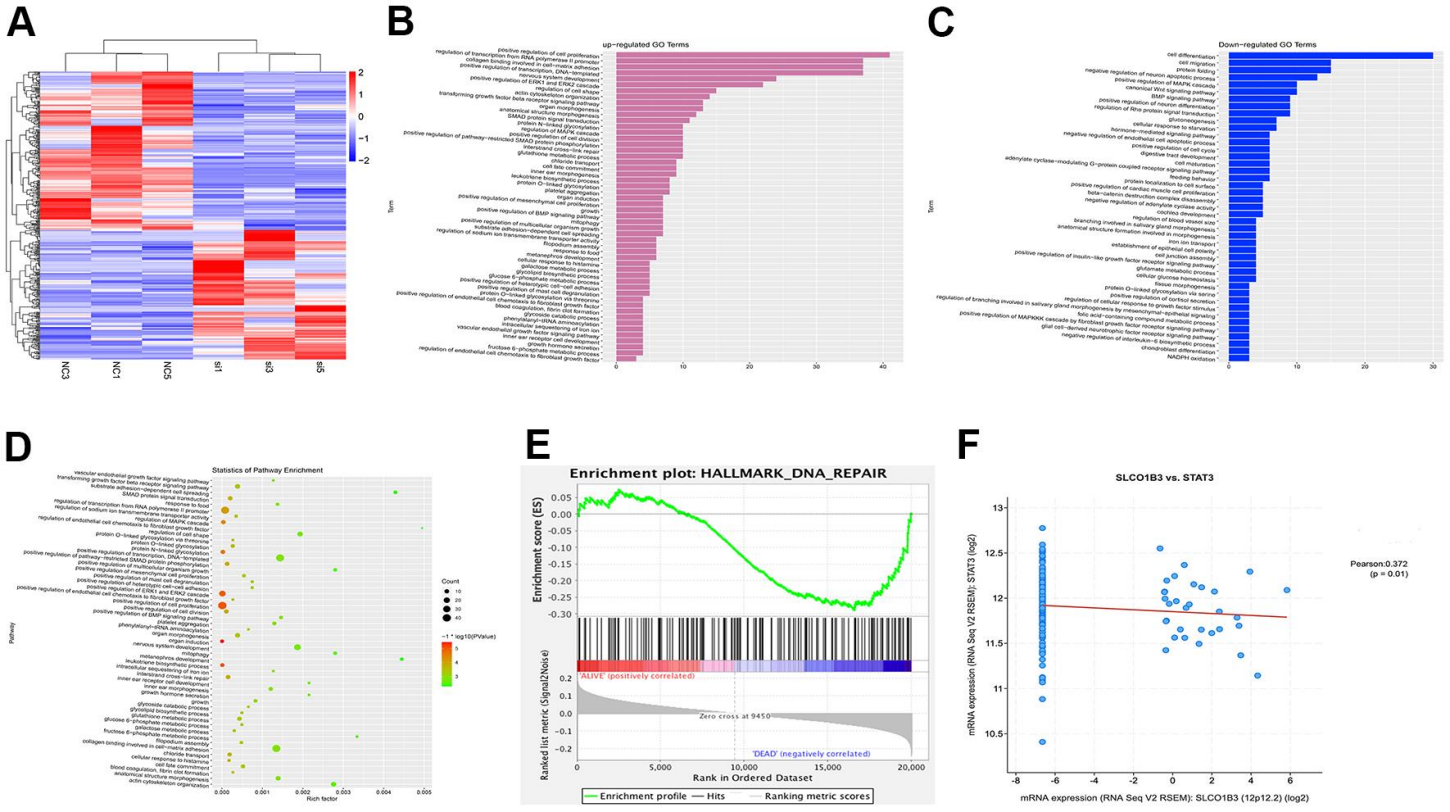
**Bioinformatics analysis of GSE163396.** Microarray data analysis was performed to investigate the molecular mechanisms underlying the function of *SLCO1B3*. (**A**) Heatmap of 286 differentially expressed genes from GSE163396 dataset (with 125 highly expressed genes and 161 lowly expressed genes). (**B**–**D**) GO functional enrichment and KEGG pathway analysis were performed based on DEGs from GSE163396 dataset. Partial results of the upregulated GO pathways were shown in panel **B**, the downregulated GO pathways were shown in panel **C**, and the KEGG pathway was illustrated in panel **D**. (**E**) Gene Set Enrichment Analysis (GSEA) revealed that most DEGs associated with the STAT3 signaling pathway were enriched in the *SLCO1B3* gene. (**F**) The co-expression analysis revealed a positive association between the *SLCO1B3* and STAT3 activation.

To investigate the molecular mechanisms underlying the function of *SLCO1B3*, we used next-generation sequencing to study the effects of *SLCO1B3* knockdown on the gene expression profile of CRC cells. A total of 286 mRNAs were differentially expressed in *SLCO1B3*-silenced HCT116 cells compared with the mRNAs in control HCT116 cells. Among which 125 were upregulated, and 161 were downregulated (Figure 5C). The mRNA expression data from this study are available from the GSE163396 dataset of the GEO database (Figure 5A). The GO functional enrichment analysis (Figure 5B, 5C), KEGG pathway analysis (Figure 5D), and GSEA (Figure 5E) revealed that many of these DEGs are involved in cancer invasion, metastasis, and DNA repair.

**Figure 6:**
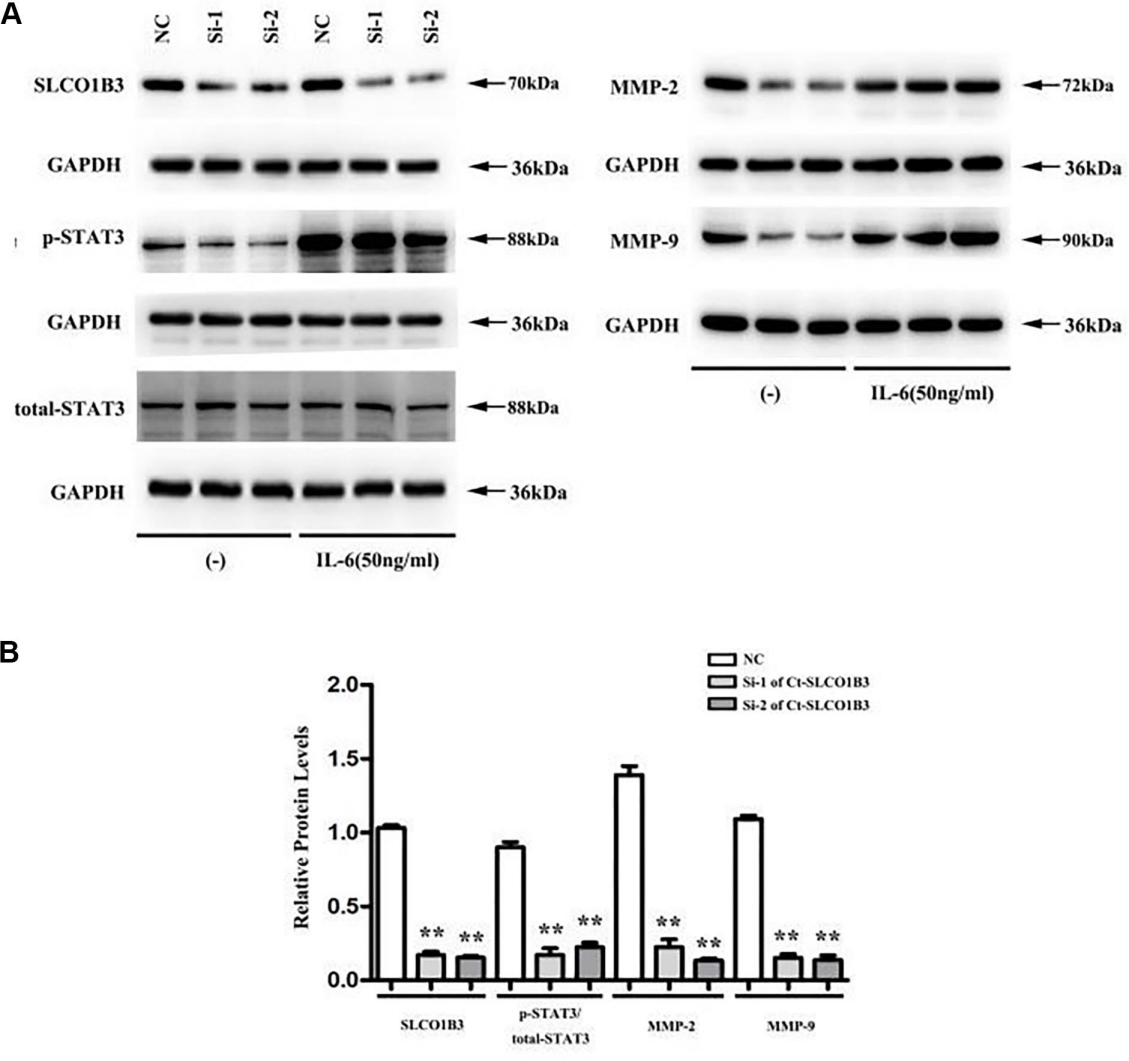
***Ct-SLCO1B3* knockdown in HCT116 cells downregulates p-STAT3, MMP-2, and MMP-9.** HCT116 cells were transiently transfected with *si1-Ct-SLCO1B3*, *si2-Ct-SLCO1B3*, or si-NC with or without IL-6 stimulation (50 ng/mL). The protein levels of *Ct-OATP1B3*, p-STAT3, total STAT3, MMP-2, and MMP-9 were determined by western blot analysis. (**A**) Gel image. (**B**) Quantified protein levels without IL-6 stimulation. n=3, **P < 0.01 vs. si-NC. NC=si-NC, Si-1=*si1-Ct-SLCO1B3*, Si-2=*si2-Ct-SLCO1B3*.

**Figure 7:**
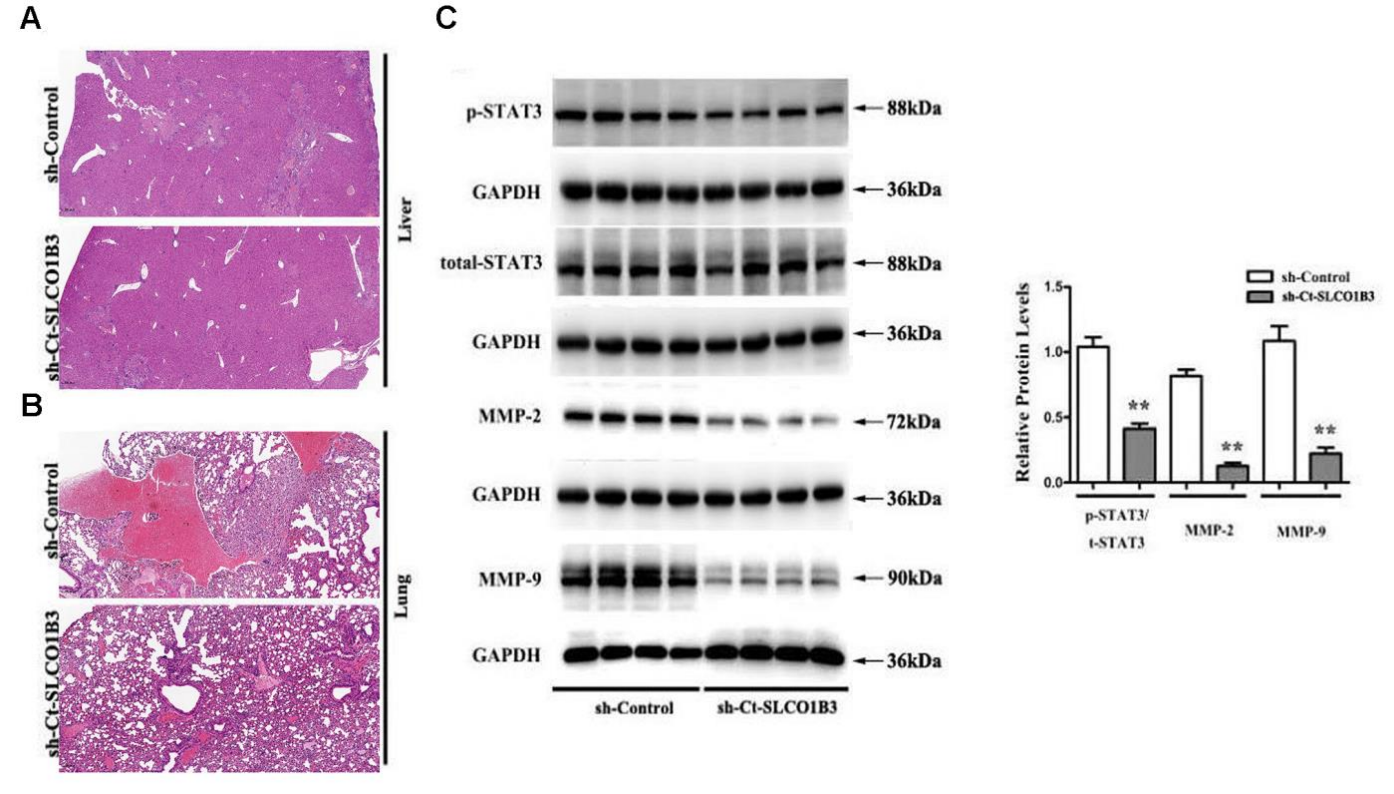
**The effects of *Ct-SLCO1B3* knockdown on CRC metastasis *in vivo*.** (**A**, **B**) H&E staining of the liver and lung tissues showing metastatic nodules. (**C**) The expression of p-STAT3, STAT3, MMP-2, and MMP-9 in metastatic tumors by western blot analysis. **P < 0.01 vs. sh-Control.

In a previous study, we found that STAT3, an important driver for CRC tumorigenesis and metastasis [7–9], was overexpressed in human CRC tissues compared with adjacent non-tumor colorectal tissues [10]. The co-expression analysis revealed a positive association between the two (Figure 5F). In our *in vitro* experiments, *SLCO1B3* knockdown in HCT116 cells downregulated p-STAT3 (P < 0.01, Figure 6A, 6B), while total STAT3 remained unchanged. This indicated that *SLCO1B3* activates the STAT3 pathway by promoting STAT3 phosphorylation. MMP-2 and MMP-9 are downstream targets of STAT3 that play critical roles in CRC invasion and metastasis [11]. *SLCO1B3* knockdown also decreased MMP-2 and MMP-9 (P < 0.01). Thus, the pro-CRC properties of *SLCO1B3* were at least partially mediated by STAT3 and its downstream mediators such as MMP-2 and MMP-9. Interleukin-6 (IL-6), a cytokine released from CRC cells, activates STAT3 to drive CRC pathogenesis and metastasis [12]. p-STAT3, MMP-2, and MMP-9 downregulated by *SLCO1B3* silencing were restored by IL-6 (Figure 6A). Female BALB/c nude mice received HT29 cells with stable *SLCO1B3* knockdown *(sh-SLCO1B3)* or an equal number of negative controls HT29 cells (sh-Control) by injection into the tail vein (n=6 per group). The mice were euthanized 21 days after the injection (Figure 7A, 7B). H&E staining of the liver Figure 7A and lung Figure 7B tissues showing metastatic nodules. The expression of p-STAT3, STAT3, MMP-2, and MMP-9 in metastatic tumors by western blot analysis (Figure 7C). **P < 0.01 vs. sh-Control.

## DISCUSSION

In the present study, the relationship between *SLCO1B3* and the clinicopathological characteristics and prognosis of CRC patients was evaluated [13]. Our bioinformatics analysis identified *SLCO1B3* as the top hub gene associated with the OS of CRC patients. Thus, *SLCO1B3* may serve as a novel therapeutic target for CRC.

In 2008, L. Wooin et al. detected *SLCO1B3* expression in most colon tumors [14]. They also found that higher *SLCO1B3* expression was associated with lower stage and lower grade tumors and improved 5-year survival within individual tumor grades. This suggested that *SLCO1B3* might exhibit antitumor properties in human CRC. However, another 2008 report showed that *SLCO1B3* overexpression in CRC cells conferred resistance to drug-induced apoptosis, supporting the protumorigenic function of *SLCO1B3* [14]. These seemingly contradictory findings were reported before the discovery of *Ct-SLCO1B3* in 2012. In 2012, Nagai and colleagues identified *Ct-SLCO1B3* in human colon and lung cancer tissues [6]. The original *SLCO1B3* was renamed *Lt-SLCO1B3* because of its enriched expression in the liver. Importantly, *Ct-SLCO1B3* was detected significantly higher than *Lt-SLCO1B3* in both human colon cancer tissues and cells [6, 15]. While *Lt-OATP1B3* (the translated product of *Lt-SLCO1B3*) was mostly found as a membrane-bound protein on the cell surface, *Ct-OATP1B3* (the translated product of *Ct-SLCO1B3*) was mainly detected in the cytoplasm of colon cancer cells [15]. In addition, *Ct-OATP1B3* and *Lt-OATP1B3* exhibited disparate patterns of post-translational modifications and proteasomal degradation [15]. Compared with *Lt-OATP1B3, Ct-OATP1B3* showed poorer transporter activity [15]. Given these differences between the two, it is reasonable to speculate that *Ct-SLCO1B3* and *Lt-SLCO1B3* may play separate roles in carcinogenesis. Herein, we reported for the first time the function of *SLCO1B3* as a carcinogen in CRC. Intriguingly, *Ct-SLCO1B3*, but not *Lt-SLCO1B3*, can be induced in response to ambient or chemical hypoxia through the HIF-1a pathway [16]. Thus, *Ct-SLCO1B3* may serve as a defense mechanism in cancer cells to sustain tumor growth under hypoxic conditions.

This study found that high *SLCO1B3* expression in human CRC tissues is associated with advanced disease stage, tumor invasion, lymph node metastasis, poor tumor differentiation, and poor OS. *SLCO1B3* knockdown effectively inhibited human CRC proliferation, migration, and invasion *in vitro* and curbed CRC tumorigenesis and metastasis *in vivo*. Mechanistically, we identified p-STAT3 and its downstream targets MMP-2 and MMP-9 as mediators of the protumorigenic function of *SLCO1B3*. The STAT3 signaling is overactivated in the CRC, and its downstream mediators such as VEGF, c-Myc and Cyclin D1, MMP-2, and MMP-9 have been linked with tumor angiogenesis, proliferation, invasion, and metastasis, respectively [17–19]. Importantly, the activation of STAT3 has been identified as a critical mechanism for resistance to 5-FU [20], the current first-line chemotherapy for advanced CRC [21]. A *SLCO1B3* inhibitor would possibly potentiate the efficacy of 5-FU in CRC by inactivating STAT3.

This study has limitations: (i) The primers designed in this study do not specifically recognize *CT-OATP1B3, LT-OATP1B3*; (ii) Immunohistochemistry in this study is also lacking. Thus, in a future study on this topic, immunohistochemistry should be performed with the antibody that recognizes *Ct-OATP1B3*.

In summary, we identified *SLCO1B3* as a novel carcinogen in the CRC that drives CRC tumorigenesis and metastasis. *SLCO1B3* inhibitors, alone or in combination with current drugs, may have therapeutic benefits in the CRC.

## MATERIALS AND METHODS

### Human tissue samples

A total of 96 pairs of tumor and adjacent normal tissues were collected between 2012 and 2014 from 96 CRC patients in the Fourth Affiliated Hospital of Hebei Medical University (Shijiazhuang, Hebei, China). The adjacent normal tissues were at least 5 cm away from the tumor edge. The clinicopathological characteristics of the patients are shown in Table 1. This study was approved by the Ethics Committee of the Fourth Affiliated Hospital of Hebei Medical University, China. All patients included in this study provided written informed consent before test-specific implementation process.

### Quantitative real-time PCR (qRT-PCR)

Total RNA was extracted using TRIzol Reagent (Thermo Fisher, USA). Complementary DNA (cDNA) was synthesized using the Reverse Transcription System (Promega, USA), following the manufacturer's instructions. Quantitative PCR (qPCR) was performed on a 7500 RT-PCR System (Applied Biosystems, USA) with the qPCR Mix (Promega, USA). The primers used in the amplification were: GAPDH, forward 5'-GGACCTGACCTGCCGTCTAG-3' and reverse 5'-GTAGCCCAGGATGCCCTTGA-3'; *SLCO1B3*, forward 5'-ACAGCAGAGTCAGCATCTTCAG-3' and reverse 5'-ATCACAAGCAAATTTCCAATTT-3'. The relative expression of *SLCO1B3* was calculated using the 2-ΔΔCt method. Each experiment was performed in triplicate.

### Cell culture

The SW480, SW620, HT29, HCT116, and RKO human CRC cell lines were purchased from the Type Culture Collection of the Chinese Academy of Science (Shanghai, China). The NCM460 normal human colon epithelial cell line was obtained from INCELL (USA). All cell lines were authenticated with short tandem repeat profiling. The SW480, SW620, and HT29 cells were cultured in Dulbecco's modified Eagle's medium (DMEM; Thermo Fisher USA), and the HCT116, RKO, and NCM460 cells were cultured in RPMI-1640 medium (Thermo Fisher). All growth media were supplemented with 10% fetal bovine serum (FBS) and 1% penicillin/streptomycin. All cells used in the experiments were mycoplasma-free.

### Transient and stable transfections

HCT116 and SW480 cells were transiently transfected with an siRNA targeting *SLCO1B3* or a negative control siRNA (Invitrogen, USA) using Lipofectamine 2000 (Invitrogen, USA), following the manufacturer's instructions. This was done to evaluate the effects of SLCO1B3 knockdown, on CRC cell proliferation, migration, and invasion. The target sequences were: *si1-SLCO1B3*, 5'-GCAACAGGAGGUACCACAUTT AUGUGGUACCUCCUGUUGCTT-3'; *si2-SLCO1B3*, 5'-GGAAAUAAUUCAGUGGCAUTTAUGCCACUGAAUUAUUUCCTT-3'; *si3-SLCO1B3*, 5'-GCACUAGGUGGAAUCAUUATT UAAUGAUUCCACCUAGUGCTT-3'. The negative control (si-NC) target sequence was: 5'-UUCUCCGAACGUGUCACGUTTACGUGACACGUUCGGAGAATT-3'. in which TT is the tail, playing as a stabilizing role. HCT116 and HT29 cells were stably transfected with a lentiviral vector carrying an shRNA targeting *SLCO1B3* (*sh-SLCO1B3:* 5'-GGAAAUAAUUCAGUGGCAUTTAUGCCACUGAAUUAUUUCCTT-3'.) or a scrambled shRNA that served as negative control (sh-Control). This was done to evaluate the effects of *SLCO1B3* knockdown, on CRC xenograft tumor growth and metastasis. Both *sh-SLCO1B3* and sh-Control were obtained from Shanghai Genechem Co., Ltd., (Shanghai, China). *SLCO1B3* knockdown in transient and stable transfections was confirmed by qRT-PCR and western blot analysis.

### CRC mouse xenograft model

Female BALB/c nude mice (five weeks old) were purchased from Beijing Vital River Laboratory Animal Technology (Beijing, China). The mouse model was established following the previously published protocol [22]. The mice were randomly assigned to two groups (n=6 per group) to receive HCT116 cells with stable *SLCO1B3* knockdown or an equal number of negative controls HCT116 cells by subcutaneous injection into the front flank. This was done to evaluate the effects of SLCO1B3 knockdown, on CRC xenograft tumor growth. Tumor length and width were measured with an external caliper once every three days. Tumor volume was calculated using the formula: tumor volume = (length × width^2^)/2. The mice were euthanized 21 days after the inoculation, and the tumors were harvested, photographed, and weighed. The mice were randomly assigned to two groups (n=6 per group) to receive HT29 cells with stable *SLCO1B3* knockdown or an equal number of negative controls HT29 cells by injecting them into the tail vein. This was done to evaluate the effects of *SLCO1B3* knockdown, on CRC metastasis. The mice were euthanized 21 days after the injection.

The lung and liver tissues were harvested and examined with hematoxylin and eosin (H&E) staining. The metastatic tumors were collected for western blot analysis. All animal experiments were performed following the international standards-3R principle of animal welfare and with permission of the Experimental Animal Ethics Committee of The Fourth Affiliated Hospital of Hebei Medical University.

### H&E staining

H&E staining was conducted following the previously published protocol [23]. The paraffin sections of the mouse liver and lung tissues were dewaxed and stained with hematoxylin (Servicebio, Wuhan, China) for 3-5 minutes. After differentiation, bluing, and dehydration in 85% and 95% alcohol for 5 minutes each, the sections were stained with eosin (Servicebio) for 5 minutes, dehydrated, sealed, and observed under the microscope.

### Western blot analysis

Western blot analysis was performed as previously described [24]. The primary antibodies used in the experiments included an anti-*OATP1B3* antibody from Proteintech Group Inc., (Wuhan, China; Catalog # 66381-1) and anti-STAT3 (Catalog # ab68153), anti-p-STAT3 (S727) (Catalog # ab32143), anti-MMP-2 (Catalog # ab92536), and anti-MMP9 (Catalog # ab58803) antibodies from Abcam (USA). Each experiment was performed in triplicate.

### Cell proliferation assay

SW480 and HCT116 cells transiently transfected with *si1-SLCO1B3*, *si2-SLCO1B3*, or si-NC were seeded in 96-well plates at a density of 3,000 cells/well and cultured for three days. Cell viability was evaluated using a 3-(4,5-dimethylthiazol-2-yl)-5-(3-carboxymethoxyphenyl)-2-(4-sulfophenyl)-2H-tetrazolium, inner salt (MTS) assay kit (Promega), according to the manufacturer's instructions. Each experiment was performed in triplicate.

### Scratch-wound healing and transwell assays

The cell migration ability was assessed using the scratch-wound healing assay [25]. The width of the wound bed was recorded at 0, 24, and 48 hours after the scratch wound. In addition, the cell migration and invasion abilities were evaluated using the Transwell migration and invasion assays, respectively. In brief, 1.5 × 10^5^ cells were loaded into the upper chamber of Transwell inserts with an 8-μm pore membrane (Corning Costar, USA), either uncoated (for migration assessment) or pre-coated with Matrigel, BD, USA, (for invasion assessment). After 48 hours, the cells that had migrated to the lower surface of the membrane were fixed, stained with crystal violet, and counted under an inverted light microscope. Each experiment was performed in triplicate.

### Identification of differentially expressed genes (DEGs) by microarray mRNA analysis

The microarray mRNA analysis was performed on HCT116 cells transfected with *si1-SLCO1B3* or si-NC. The raw microarray data were converted to gene expression data using the Edge R package [26]. The DEGs (|logFC|>2 and padj<0.05) were identified with the Limma R package.

### GSEA, GO, and KEGG analysis of DEGs

The Gene Set Enrichment Analysis (GSEA) was performed using the Category package (version 2.10.1). The functional enrichment Gene Ontology (GO) analysis and the Kyoto Encyclopedia of Genes and Genomes (KEGG) pathway analysis were performed with the DAVID online tools. Fisher's exact test was used to select the significant pathways. A P value less than 0.05 was considered statistically significant.

### Bioinformatics analysis

The overall survival (OS) and mRNA expression data of CRC patients were retrieved from the GSE123734 dataset from the GEO (Gene Expression Omnibus) database. The relationship between OS and *SLCO1B3* expression was analyzed by the Kaplan–Meier method. The CytoHubba application in Cytoscape was used to identify and rank the hub genes. The Cytoscape software was used to generate the protein-protein interaction diagram.

### Statistical analysis

Each experiment was performed in triplicate. The results are presented as mean ± standard deviation. The differences between the two groups were evaluated using the two-tailed Student's t-test. The correlation between tumorous *SLCO1B3* and the clinicopathological characteristics and prognosis of CRC patients was evaluated using the chi-square test. The Kaplan–Meier method (log-rank test) was used to interpret the survival curves. All statistical analyses were performed using the GraphPad Prism 5.0 and SPSS 21.0 software. A P-value of less than 0.05 was considered statistically significant.

### Data availability statement

The mRNA expression data from this study will be deposited in the GSE163396 dataset of the GEO database.
